# Consequences of a Refuge for the Predator-Prey Dynamics of a Wolf-Elk System in Banff National Park, Alberta, Canada

**DOI:** 10.1371/journal.pone.0091417

**Published:** 2014-03-26

**Authors:** Joshua F. Goldberg, Mark Hebblewhite, John Bardsley

**Affiliations:** 1 Wildlife Biology Program, Department of Ecosystem and Conservation Science, College of Forestry and Conservation, University of Montana, Missoula, Montana, United States of America; 2 Department of Mathematics and Statistics, University of Montana, Missoula, Montana, United States of America; National Research Council of Italy (CNR), Italy

## Abstract

Refugia can affect predator-prey dynamics via movements between refuge and non-refuge areas. We examine the influence of a refuge on population dynamics in a large mammal predator-prey system. Wolves (*Canis lupus*) have recolonized much of their former range in North America, and as a result, ungulate prey have exploited refugia to reduce predation risk with unknown impacts on wolf-prey dynamics. We examined the influence of a refuge on elk (*Cervus elaphus*) and wolf population dynamics in Banff National Park. Elk occupy the Banff townsite with little predation, whereas elk in the adjoining Bow Valley experience higher wolf predation. The Banff refuge may influence Bow Valley predator-prey dynamics through source-sink movements. To test this hypothesis, we used 26 years of wolf and elk population counts and the Delayed Rejection Adaptive Metropolis Markov chain Monte Carlo method to fit five predator-prey models: 1) with no source-sink movements, 2) with elk density-dependent dispersal from the refuge to the non-refuge, 3) with elk predation risk avoidance movements from the non-refuge to the refuge, 4) with differential movement rates between refuge and non-refuge, and 5) with short-term, source-sink wolf movements. Model 1 provided the best fit of the data, as measured by Akaike Information Criterion (AIC). In the top model, Banff and Bow Valley elk had median growth rates of 0.08 and 0.03 (95% credibility intervals [CIs]: 0.027–0.186 and 0.001–0.143), respectively, Banff had a median carrying capacity of 630 elk (95% CI: 471.9–2676.9), Bow Valley elk had a median wolf encounter rate of 0.02 (95% CI: 0.013–0.030), and wolves had a median death rate of 0.23 (95% CI: 0.146–0.335) and a median conversion efficiency of 0.07 (95% CI: 0.031–0.124). We found little evidence for potential source-sink movements influencing the predator-prey dynamics of this system. This result suggests that the refuge was isolated from the non-refuge.

## Introduction

Spatial refugia have many potential impacts on predator-prey dynamics, including promoting stability through spatial structure, creating dynamic fluctuations due to the way in which prey use refuges [1]–[3] and interact with predators [4], [5], or producing instability (local extinction) via complex spatiotemporal interactions [6]. Previous studies have considered the consequences of spatial refugia through theoretical models [7]–[10], laboratory experiments [11], [12] and field observation [13], [14]. These examinations generally suggest that spatial refugia stabilize the predator-prey dynamics of single populations of predators and prey, leading to increased persistence of predators and prey, as well as increased abundance of prey [2], [15] and under certain conditions predators [1], [3]. Despite this widespread agreement, refugia do not necessarily lead to the stable coexistence of predators and prey [16], [17]. The sources of these divergent outcomes and the interactions between refuge and non-refuge populations in empirical systems remain poorly understood.

Refugia have often been related to spatial heterogeneity in immigration/emigration rates of prey, but also imply spatial heterogeneity in predation. Harvested populations frequently show distributional shifts in response to predation, whereby individuals from surrounding areas shift their habitat use to take advantage of available resources once used by harvested individuals [18]–[20]. Within the landscape context, refugia may serve as source habitats, supporting self-sustaining populations with net immigration, while areas of predator-prey co-occurrence may serve as sink habitats, relying on net emigration to maintain prey populations [21], [22]. Conversely, non-refuge habitat may provide a source of emigrants to refugia. In this case, refugia support large populations in the absence of predation or may serve as sinks if habitat is of poor quality. Thus, spatial refugia in predator-prey interactions may foster source-sink dynamics in both prey and predators across a landscape. Source-sink movements between hunted populations and populations with little or no hunting have been widely inferred based upon densities, demographic composition and/or genetics [20], [23], [24], but these results have not held in systems with natural predation [25]. Moreover, while many invoke predation as a probable factor in source-sink dynamics [26], [27], population dynamics consequences of source-sink movements on predator populations have seldom been explored. Refugia may impact predator populations within the broader landscape context.

Wolves and their prey may provide an important applied system for examining these predator-prey source-sink dynamics. The recovery of wolves in western North America [28] has often resulted in spatial heterogeneity in the populations of gray wolves (*Canis lupus*) and their prey, such as elk (*Cervus elaphus*) or deer (*Odocoileus* spp.). Refugia have frequently developed because of the avoidance relationship between wolves and humans [29], [30]. In areas with high human activity or development, wolf survival and populations decline, leading to reduced predation on ungulate prey [31], and the development of refugia for species such as deer and elk [32]–[35]. Prey refugia may also develop naturally due to the manner in which wolves exploit their prey base [36]. These prey refugia create the potential for source-sink dynamics to develop among prey sub-populations via movements between centers of human activity and less developed areas [20], [37]. Prey dispersal from human-created sources to neighboring areas may not increase prey populations in these sinks, but instead buoy wolf populations. Similarly, wolves may make temporary movements to hunt large prey populations at the edges of developed refugia, leading to increased wolf numbers and declines in the more vulnerable prey outside the refuge. These potential source-sink dynamics take on particular importance in the context of managing prey refugia, as high ungulate prey density poses threats to economic activity, human health and ecosystem health [35], [38], [39]

An example of this potential source-sink interaction between wolves and prey with a human-induced refuge occurs in Banff National Park (BNP). Wolves recolonized BNP through dispersal in the mid 1980's, but avoided the townsite of Banff, resulting in differential predation by wolves on elk [31], [40], [41]. In the Bow Valley, adjacent to Banff, wolves prey primarily upon elk [42], whereas the Banff elk population exists with little or no known predation by wolves [39]. Within 15-years, the distribution of elk shifted to where most elk in the valley occupied the predation refuge surrounding the townsite [39]. This distributional shift may lead to source-sink movements between the refuge and non-refuge areas with the potential to influence the overall predator-prey dynamics of BNP. The townsite of Banff may function as an elk refuge from wolf predation and affect the predator-prey dynamics of the Bow Valley wolf-elk system. This wolf-elk dynamic represents an interesting predator-prey refuge system to examine source-sink interactions in a spatially heterogeneous environment. Moreover, these spatial trophic interactions take on applied importance within the context of the conservation mandate of a National park, because the human-created refuge may alter the predator-prey dynamics of adjacent “natural” areas.

The development of a spatial predation refuge in and around the townsite of Banff provides an opportunity to test source-sink movements between Banff and Bow Valley of elk or wolves and the impacts of these movements on predator-prey dynamics of the system. We hypothesized four ways in which the Banff refuge might influence the Bow Valley predator-prey dynamics through source-sink dynamics in the prey (elk), predator (wolves), or both. First, since the Banff elk existed at or near carrying capacity for much of the time-series [39], [43], this population likely experienced density-dependent competition for resources, which may have motivated elk dispersal to neighboring areas, such as the Bow Valley. Thus, the Banff townsite may have served as a source elk population for the adjacent Bow Valley through density-dependent elk dispersal from source to sink. Alternatively, Bow Valley elk may move to Banff to avoid wolf predation, such that the Bow Valley serves as an elk source population, provided emigration rate exceeds mortality rate (i.e., predator avoidance source-sink dynamics). Third, these two kinds of movements may occur simultaneously, such that elk disperse from Banff to Bow Valley in a density-dependent fashion at the same time as Bow Valley elk move to Banff to escape wolf predation. Finally, wolves may make short-term, temporary movements to prey on the Banff elk directly (without elk dispersal). This mechanism implies no source-sink phenomenon for prey, but rather source-sink movements for wolves that allow Banff to act as a source of elk for Bow Valley wolves. These predator source-sink movements occur on a short-term basis. Of course, these mechanisms may occur in parallel, as in the third hypothesis, or other forms of wolf-elk source sink dynamics may occur (e.g., both movements of elk and wolves), but we started with these four hypotheses based on previous studies, in addition to a fifth null model with completely independent sub-populations. We assess these potential interactions between the Banff refuge and the Bow Valley with these five predator-prey models. We fit competing continuous-time predator-prey models to time-series counts of wolves and elk in the refuge and non-refuge populations from a 26-year period. We fit time-series models using the novel Delayed Rejection Adaptive Metropolis (DRAM) Markov chain Monte Carlo (MCMC) method [44].

## Materials and Methods

### Study Area

BNP is located on the eastern front of the Canadian Rocky Mountains. The rugged terrain gives rise to a climate characterized by long, cold winters with short, irregular warm periods, and short, dry summers. The Bow Valley (181 km^2^) and area surrounding the Banff townsite (41 km^2^) have been previously partitioned into distinct ecological zones based upon variation in human, wolf and elk densities [39], [43]. Elk populations correspond to these zones, showing strong home range site fidelity and rarely relocating to adjacent zones [45], [46]. Home range size of elk in Banff was 28 km^2^ [46], [47]. Neither Banff nor Bow Valley elk migrate to distinct seasonal ranges [46], so seasonal counts appropriately describe the population dynamics of Banff and the Bow Valley. Hebblewhite et al. [43] provide further description of the study area.

Parks Canada monitored wolf and elk winter populations annually in BNP during the study period over 26 years from 1985–1986 (i.e., November 1985 to April 1986) through 2010–2011 [48]. The agency began counting the Bow Valley (non-refuge) wolf population upon wolf recolonization in the winter of 1985–1986 [43], [49]. Prior to the winter of 1992–1993, the Bow Valley supported two wolf packs (the Castle and Spray packs), which then merged to form a single pack (the Bow Valley pack). We summed the population counts from the Castle and Spray packs to determine the total number of wolves in the Bow Valley during the early years of the study period. Wolves preyed primarily upon elk, which made up to 70% of wolf diet, within our study area over the time series. Secondary prey species included caribou (*Rangifer tarandus*), mule deer (*Odocoileus hemionus*), white-tailed deer (*Odocoileus virginanus*), moose *(Alces alces*) and bighorn sheep (*Ovis canadensis*), although wolves rarely interacted with these species due to rarity or spatial segregation [42]. Wolf diet composition justifies a predator-single prey approximation of population dynamics [50].

Parks Canada conducted late-winter aerial surveys to determine elk populations in the town of Banff (refuge) and the Bow Valley [43], [49]. We applied a sightability adjustment of 13% to correct observer bias in the elk population counts [49]. Parks Canada took aggressive management actions to control growing urban elk populations in the Banff townsite (Banff hereafter) starting in 1998. From 1998 to 2001, Parks Canada relocated elk far outside the system (equivalent to harvest with no return) to mitigate emerging human-elk conflicts [51]. As a part of this management plan, Parks Canada began an aversive conditioning program to further combat the problems of habituated elk [47]. Outside of this three-year period, Banff elk have not been subjected to any human harvest under the management authority of Parks Canada. Similarly, the Bow Valley wolf and elk populations have not experienced any human hunting or culling for the duration of our study, although both were subjected to occasional vehicle caused mortality despite extensive highway mitigation [52].

### Source-Sink Modeling

**Figure 1:**
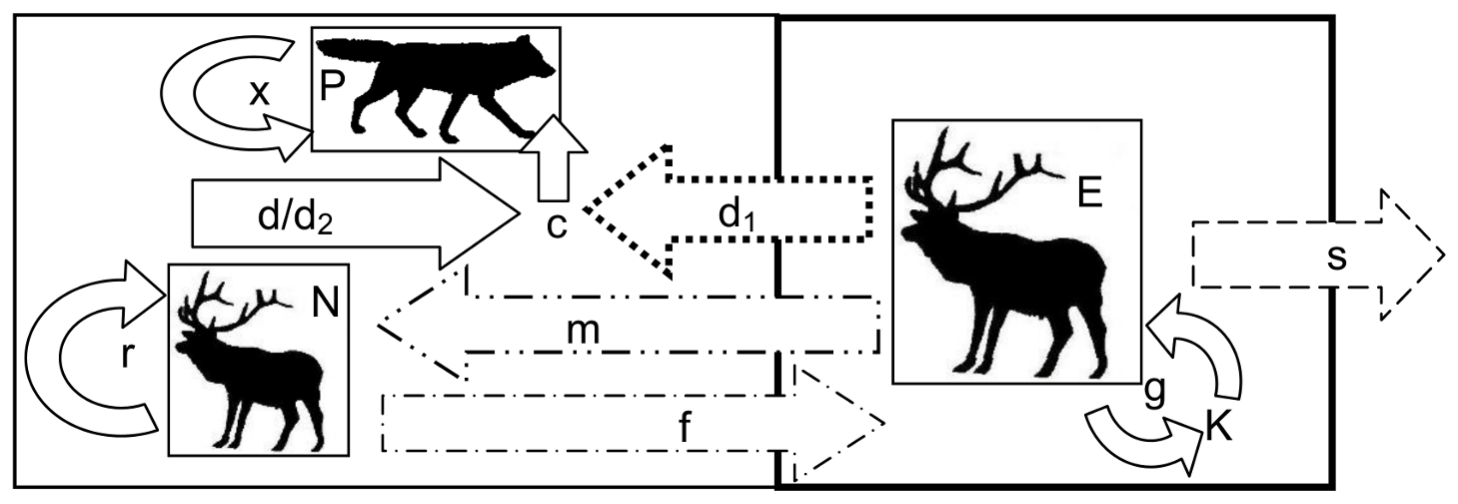
Diagram of the modeled predator-prey dynamics. Schematic diagram showing the modeled predator-prey interactions of Banff elk (E), Bow Valley elk (N) and Bow Valley wolves (P) for Models 1, 2, 3, 4 and 5. Arrows with solid lines represent interactions present in all years in all models. The Banff elk grow logistically with growth rate, g, and carrying capacity, K. The Bow Valley elk grow exponentially with rate, r, and encounter or interact with wolves at rate, d or d_2_. Bow Valley wolves convert some proportion of elk encountered into new wolves with conversion efficiency, c, and have mortality rate, x. The dashed arrow (— —) represents the Banff elk relocation parameter (s) that occurred during the years 1998–2001 in all models. The dashed and double dotted arrow (– ·· –) represents the density-dependent dispersal parameter (m) for Models 2 and 4, the dashed and single dotted arrow (– · –) represents the anti-predator movement parameter (f) for Models 3 and 4, and the dotted arrow (▪▪) represents the short-term, source-sink wolf predation parameter (d_1_) for Model 5.

We tested for the various mechanisms of source-sink dynamics described above by fitting five predator-prey models that considered: (i) null model of completely separate Banff and the Bow Valley wolf-elk systems, (ii) density-dependent elk dispersal from Banff to the Bow Valley, (iii) elk predation risk avoidance movements from Bow Valley to Banff, (iv) both density-dependent elk dispersal from Banff to the Bow Valley and predation avoidance movements from Bow Valley to Banff, and (v) short-term, source-sink wolf movements (Fig. 1). Although the data consist of discrete (annual) realizations of both continuous and discrete processes acting on the populations, we chose continuous model formulations. Discrete models describe systems, where reproduction, mortality and species interactions occur in short, segregated time-intervals, whereas continuous models represent these factors as on-going processes influencing population dynamics [53]. These different representations have been shown to produce different deterministic dynamics and stability conditions [53], [54]. We selected a continuous model framework, since we wanted to best capture the continuous dispersal and predation terms of interest, while maintaining the elegance and interpretability of the parameters. While we recognize that reproduction in our study species occurs in discrete, annual events, the continuous forces may play a more important role in structuring the population dynamics and source-sink phenomena of interest [54]. When modeling coupled wolf-elk dynamics, we used the Lotka-Volterra model, which despite its theoretical and mechanistic shortcomings [55], has proven useful in previous analyses of wolf-prey dynamics [56]. We find this approach to represent a compromise between over-simplifying the many complex processes acting on these populations, and over-specifying models with reduced interpretability and support from the available data. We aimed to produce a useful caricature of the system that allows us to assess the relative support for the potential source-sink mechanisms described before adding complexity.

### Null Model 1: Independent Elk and Wolf Populations

The first continuous-time predator-prey model considers the Banff elk (*E*) population, and Bow Valley wolf (*P*) and elk (*N*) populations separately, a sort of null model. This model assumed no elk dispersal between populations and no wolf predation on Banff elk. We fit the Banff elk population with a density-dependent (logistic) growth model:
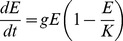
(1)The parameter, *g*, gives the per capita elk population growth rate, and *K* represents the carrying capacity of the Banff townsite. We used an additional model parameter, *s*, to describe the elk during the active management plan (1998–2001):
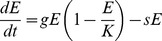
(2)The parameter, *s*, indicates the proportion of the elk population relocated annually. We modeled the Bow Valley wolves and elk with the Lotka-Volterra equations:
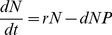
(3)

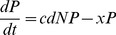
(4)The parameter, *r*, is the (exponential) per capita growth rate of the Bow Valley elk, *d* represents the interaction (or encounter) rate between the wolves and elk, *c* represents the conversion efficiency of the wolves, and *x* gives the wolf death rate [57], [58]. In this model, Banff and the Bow Valley do not interact and no source-sink dynamics are considered.

### Model 2: Density-dependent Elk Dispersal

We used a different set of equations to describe a system with net elk dispersal from Banff (putative source) to the Bow Valley (sink). Before the management actions of Parks Canada, we used the equations:
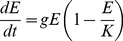
(5)

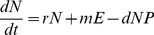
(6)

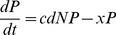
(7)The new term, *mE*, shows that some proportion of Banff elk move from Banff to the Bow Valley each year. This dispersal constant, *m*, is contained implicitly within the growth rate of the Banff elk, *g*, which accounts for birth, death and dispersal in this model. We only used this dispersal term before the relocation efforts of the park service, because relocation released the Banff elk from the pressures of density-dependent competition, removing the likely stimulus for dispersal to the Bow Valley [59]. As in equation (2), we used the relocation term (-*sE*) to model the effect of the aggressive management efforts on the Banff elk population from 1998 to 2001.

### Model 3: Predation Avoidance Movement

We used a different set of equations to describe a system with net elk dispersal from Bow Valley (source) to Banff (sink) to avoid predation risk. Before the management actions of Parks Canada, we used the equations:
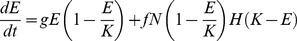
(8)

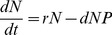
(9)

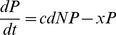
(10)The new term, *fN(1-E/K)*, shows that some proportion of Bow Valley elk move from Bow Valley to Banff each year in a density dependent fashion, i.e. fewer elk will move into Banff as the elk population approaches carrying capacity, *K*. We incorporated this new term conditionally with respect to the ratio, *E*/*K*, such that this density-dependent movement from Bow Valley to Banff only occurred when Banff Elk did not exceeded carrying capacity (*E*/*K*≤1). We implemented this condition by multiplying the movement term by the Heaviside function, *H(K-E)*, where *H*(*x*) = 1 for *x*≥0 and *H(x)* = 0 for x<0. This Bow Valley elk anti-predator movement constant, *f*, is contained implicitly within the growth rate of the Bow Valley elk, *r*, which accounts for birth, death and emigration in this model. We implemented this elk risk avoidance term in all years, since elk may have fled wolf predation in the Bow Valley throughout the duration of the study. As in (2), we used the relocation term (-*sE*) to model the effect of the aggressive management efforts on the Banff elk population from 1998 to 2001.

### Model 4: Differential Elk Movement

We synthesized the novel terms from Model 2 and Model 3 into a single system with elk dispersal from Banff to the Bow Valley and with elk predation avoidance movements from Bow Valley to Banff to avoid predation. Thus, elk could move in either direction between Banff and the Bow Valley at different rates. Prior to the elk relocation by Parks Canada, we used the equations:
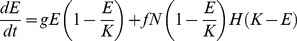
(11)

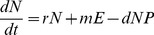
(12)

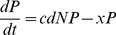
(13)As before, the dispersal represented by *mE* occurs only before the management undertaken by Park Canada, and the term, *fN(1-E/K)*, only operates when Banff Elk exist below carrying capacity (*E*/*K*≤1) through the use of the Heaviside function as in Model 3. As in equation (2), we used the relocation term (-*sE*) to model the effect of the aggressive management efforts on the Banff elk population from 1998 to 2001.

### Model 5: Short-term, Source-Sink Wolf Movement

We fit a fifth model to describe a system with short-term, source-sink type wolf movements between the refuge and Bow valley, which manifested as differential encounter rates between wolves and elk in the Bow Valley or Banff refuge. We modeled this system with the equations:
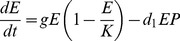
(14)

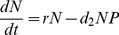
(15)

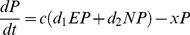
(16)The interpretation of the parameters remains unchanged from previous models. This model incorporates two different interaction rates between wolves and Banff elk (*d_1_*), and wolves and Bow Valley elk (*d_2_*). We compared these encounter rates to determine the relative contribution of each of the elk populations to the wolf population dynamics. As in Model 1, equation (2), we used the relocation term (-*sE*) to model the effect of relocation on the Banff elk population from 1998 to 2001.

### Bayesian Population Modeling

We fit these competing predator-prey models to the population count data using MATLAB [60]. We used a two-step process to estimate the parameters corresponding to the best-fit of the above five models to the data. First, for given parameter values, we solved the differential equations numerically. Secondly, we estimate the parameters that correspond to the best model fit of the data in the least squares sense using an optimization method [61]. This nonlinear regression approach may converge to local minima, or not converge at all, if given poor initial parameter values [61]. However, even in the event that good parameter estimates are obtained, which in our examples was always the case, classical techniques for obtaining confidence intervals and/or variance estimates for the parameter values require the linearization of the nonlinear population models.

We instead use Bayesian MCMC methods to provide robust parameter estimates and assess the uncertainty in the estimated parameters [44], [61], [62]. In short, MCMC generates samples of the unknown parameters, which are distributed according to the so-called posterior probability distribution given by Bayes' Law. The samples are obtained by computing random draws from a proposal distribution, which are then accepted with a probability defined by the ratio of the likelihood function evaluated at the proposed and most recently accepted samples. MCMC techniques offer many potential advantages for modeling biological systems. The added flexibility inherent in MCMC allows for greater model complexity and structure, and, in many cases, for more accurate confidence bounds on the parameter estimates. Moreover, using MCMC to fit the above five models to the wolf-elk data improves the power to detect significant interactions or effects [63], and allows for the parameters to be constrained to biologically realistic or plausible values, which can improve parameter identification [44], [64].

We used the delayed rejection adaptive Metropolis (DRAM) implementation of MCMC devised by Haario et al. [44]. DRAM combines adaptive techniques, which scale the distribution of random proposed draws by the covariance of the MCMC chain [65], and delayed rejection procedures, which use multiple proposal distributions of different size, to sample the parameter distributions efficiently [66]–[68]. The combination of these methods improves the speed and efficiency at which the MCMC chain converges in distribution to the posterior distribution of the unknown parameters [44]. This algorithm was implemented in MATLAB [69] and is also available in the R package FME [70].

For each model, we solved the ordinary differential equations with a moderately stiff solver based upon an implementation of the trapezoidal rule with a free interpolant [71]. We used this solution to interpolate values at each time-step and obtain parameter estimates from a nonlinear least squares fit. We changed the population estimates to have the same scale before computing the least squares fit to ensure that the Banff elk, Bow Valley elk and wolf populations all received equal weight in the parameter estimation routine. After fitting the five models using nonlinear least squares, we used the fitted parameter estimates as initial values for DRAM MCMC analysis. We employed uniform priors for all parameters. We additionally specified lower bounds of zero inclusive for dispersal, risk avoidance and differential predation parameters and zero exclusive for all other parameters in all models. We placed an upper bound on the cull parameter of 1, since no more than the entire Banff elk population could be removed in a year and an upper bound on the Banff elk carrying capacity of 3,000, as a compromise between biological realism and limiting the inclusion of too much prior information in the model. We conducted DRAM MCMC analysis with 110,000 samples of each parameter estimate for each model, using up to three delayed rejection steps for any given sample in the chain, and adapting the covariance every one-thousand iterations. After discarding the first 10,000 samples (as so-called burn in), in which the samples from the chain may not lie in the support of the posterior distribution, we ensured that the chain had converged by examining trace plots, marginal and pair-wise parameter histograms. For converged samples, we computed the mean, median and 95% credibility intervals for all parameter estimates. We then compared the fit of the models graphically and by calculating the residual sum of squares (RSS) with the median chain parameter estimates. We used the RSS to compute AICc, Akaike's Information Criterion [72], corrected for small sample sizes, and evaluate the relative support of all five models. AICc provides a means of considering the relative support of each model by balancing the improved model fit gained by adding parameters against model parsimony. We used: AICc = *n*ln(RSS/*n*)+2*k*+[2*k*(*k*+1)]/(*n* – *k* – 1), with *n* being sample size, and *k* representing the number of parameters in a model. We then computed ΔAICc for each model *i*, as ΔAICc*_i_* = AICc*_i_* – min(AICc) [72]. With these values of ΔAICc, we calculated the Akaike weights, *w_i_*, to evaluate the relative likelihood support for each model, where *w_i_* = exp(−0.5ΔAICc*_i_*)/∑*_i_* exp(−0.5ΔAICc*_i_*) [72]. However, as others have noted, using AIC in model selection of predator-prey dynamics often favors simpler empirical models more than theoretically sound, yet more heavily parameterized models [73]. Thus, regardless of the top model form, we interpret and present results from all models, as well as parameter estimates, to guide our ecological understanding of this system.

## Results

### Null Model 1: Independent Wolf and Elk Populations

**Figure 2:**
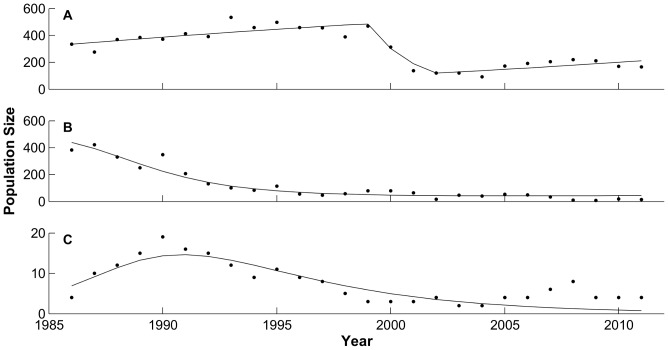
Model 1 fit for all populations. Model 1 fit for the Banff elk population (a), Bow Valley elk population (b) and Bow Valley wolf population (c) from winter of 1985/1986–2010/2011. Population data shown with dots (•) and model fit shown with a solid line (—).

Model 1 had the lowest AICc, while receiving 0.629 of the Akaike weight among candidate models (Fig. 2, Table 1, 2). For the Banff elk, the model captured the gradual increase towards carrying capacity during the first phase of the data, the rapid decline in response to the relocation effort and the resumption of population growth after the removal of this management pressure. Outside the refuge in the Bow Valley, the model captured the gradual decline of the elk population and the rise and fall of the wolf population. Banff and Bow Valley elk had median growth rates of 0.08 and 0.03 (95% credibility intervals [CIs]: 0.027–0.186 and 0.001–0.143), respectively, Banff had a median carrying capacity of 630 elk (95% CI: 471.9–2676.9), Bow Valley elk had a median wolf encounter rate of 0.02 (95% CI: 0.013–0.030), and wolves had a median death rate of 0.23 (95% CI: 0.146–0.335) and a median conversion efficiency of 0.07 (95% CI: 0.031–0.124) (Table 1).

**Table 1:** Parameter estimates and 95% credibility intervals (CIs) for the Banff elk, Bow Valley elk and Bow Valley wolves from winters of 1985/1986–2010/2011 for Models 1, 2, 3, 4 and 5.

Population	Parameter	Model 1	Model 2	Model 3	Model 4	Model 5
Banff Elk	Growth Rate (g)	0.08 (0.027, 0.186)	0.07 (0.013, 0.244)	0.05 (0.002, 0.190)	0.04 (0.001, .194)	0.07 (0.021, 0.231)
Banff Elk	Carry Capacity (K)	630 (471.9, 2676.9)	677 (419.2, 2782.3)	513 (391.2, 2143.2)	520 (401.7, 519.6)	1039 (452.1, 2871.3)
Banff Elk	Initial Pop. (E_0_)	335 (292.2, 380.1)	340 (265.4, 415.8)	298 (179.1, 408.0)	300 (190.1, 406.2)	340 (268.7, 411.3)
Banff Elk	Relocation parameter (s)	0.52 (0.399, 0.703)	0.51 (0.333, 0.811)	0.56 (0.361, 0.898)	0.56 (0.357, 0.888)	0.52 (0.340, 0.830)
Banff Elk	Dispersal Rate (m)		0.01 (0.001, 0.037)		0.01 (0.001, 0.036)	
Banff Elk	Banff Elk Encounter Rate (d_1_)					<0.01 (<0.001, 0.004)
Bow Valley Elk	Growth Rate (r)	0.03 (0.001, 0.143)	0.03 (0.001, 0.106)	0.03 (0.001, 0.118)	0.03 (0.001, 0.097)	0.04 (0.001, 0.129)
Bow Valley Elk	Encounter Rate (d or d_2_)	0.02 (0.013, 0.030)	0.02 (0.014, 0.030)	0.02 (0.013, 0.027)	0.02 (0.015, 0.029)	0.02 (0.014, 0.029)
Bow Valley Elk	Initial Pop. (N_0_)	438 (380.5, 497.2)	442 (385.4, 492.0)	438 (389.3, 488.9)	443 (394.1, 493.7)	440 (388.8, 491.6)
Bow Valley Elk	Anti-predator Movement Rate (f)			0.19 (0.012, 0.775)	0.18 (0.011, 0.639)	
Bow Valley Wolf	Death Rate (x)	0.23 (0.146, 0.335)	0.23 (0.165, 0.325)	0.23 (0.158, 0.316)	0.23 (0.163, 0.310)	0.25 (0.165, 0.427)
Bow Valley Wolf	Conversion Efficiency (c)	0.07 (0.031, 0.124)	0.07 (0.037, 0.116)	0.07 (0.037, 0.116)	0.06 (0.038, 0.101)	0.07 (0.034, 0.120)
Bow Valley Wolf	Initial Pop. (W_0_)	7 (4.6, 9.7)	6 (4.3, 8.7)	7 (4.9, 9.3)	6 (4.4, 9.0)	7 (4.7, 9.4)

**Table 2:** Model selection results for Models 1, 2, 3, 4 and 5 fit to the time-series data of Banff elk, Bow Valley elk and Bow Valley wolves for winters of 1985/1986–2010/2011.

Model	RSS^a^	AICc^b^	ΔAICc^c^	*w_i_* d
Model 1 – Separate	284.16	124.12	0.00	0.629
Model 2 – Density-dependent Elk Dispersal	287.63	127.79	3.67	0.101
Model 3 –Predation Avoidance Movement	281.66	126.15	2.03	0.228
Model 4 – Differential Elk Movement	288.50	130.82	6.70	0.022
Model 5 – Short-term, Source-Sink Wolf Movements	300.10	131.10	6.98	0.019

a RSS is the sum of the squared residuals from the model prediction with the median chain value of 100,000 MCMC samples.

b AICc is Akaike's information criterion corrected for a small sample computed based upon the RSS.

c ΔAICc is the difference between the model with the lowest AICc and a particular model.

d *w_i_* is the relative model likelihood.

### Model 2: Density-Dependent Elk Dispersal

**Figure 3:**
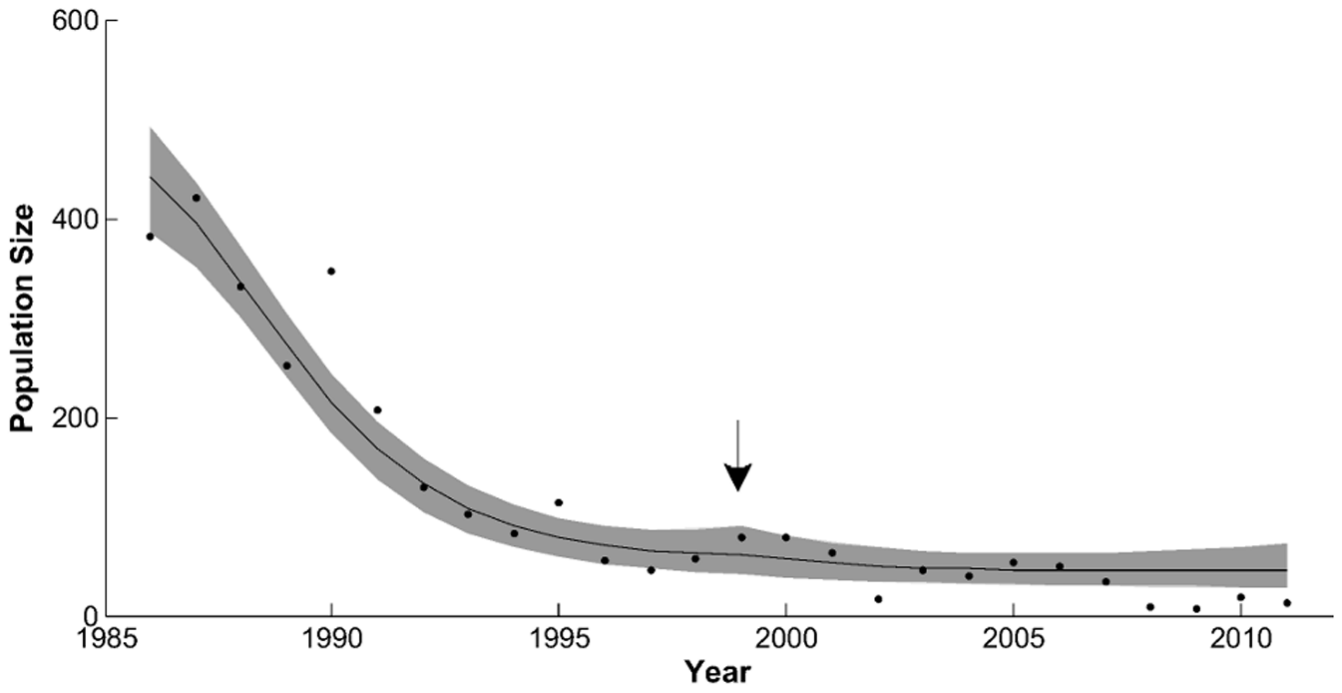
Distribution of Model 2 fit. Model 2 fit for the Bow Valley elk population from winters of 1985/1986–2010/2011. Population data shown with dots (•), the model fit shown with a line (—), the 95% credibility interval for the model distribution shown with gray band.

Model 2 had a larger RSS and AICc and received less of the likelihood weight (0.101) than Model 1 (Table 2). Despite these large differences in model selection criteria, the graphical fit of Model 2 did not differ qualitatively from Model 1, capturing the rise to carrying capacity and sharp decline in the Banff elk population, and the changes in the Bow Valley wolf and elk populations. Model 2 achieved this fit with parameter estimates similar to those of Model 1 (Table 1). A comparison of the parameters from Model 1 to Model 2 shows that none of the common parameters (all parameters but *m*) changed significantly with 95% credibility (Table 1). The additional dispersal parameter, *m*, had a median value of 0.01 with 95% CI of (0.001, 0.037) (Table 1). The migration parameter does yield a subtle change in the distribution of the model (Fig. 3). The model distribution has a bump just before the major relocation effort takes place, mirroring a slight increase in the elk population.

### Model 3: Predation Avoidance Movement

The quantitative fit of Model 3 fell between Model 1 and Model 2 by AICc, and Model 3 received a likelihood weight of 0.228 (Table 2). Estimates for comparable parameters of Model 3 did not differ significantly from the estimates of Models 1 or 2 at the 95% credibility level (Table 1). For the new parameter in this model, the Bow Valley elk anti-predator movement rate, we found a median value of 0.19 with 95% CI (0.012, 0.775). Incorporating elk risk avoidance movements to the refuge of the Bow Valley, however, did not qualitatively change the graphical distribution of the model.

### Model 4: Differential Elk Movement

The quantitative fit of Model 4 did not meet that of Model 1, Model 2 or Model 3 by AICc, and Model 4 received a likelihood weight of 0.022 (Table 2). The parameter estimates from Model 4 did not differ significantly from estimates of comparable parameters in Models 1, 2 or 3 at the 95% credibility level (Table 1). The Banff elk dispersal parameter, *m*, had a median value of 0.01 with 95% CI of (0.001, 0.036), similar to Model 2, which shared this term (Table 1). For the Bow Valley elk anti-predator movement rate, *f*, we found a median value of 0.18 with 95% CI (0.011, 0.639), which did not differ from the parameter estimate from Model 3 at the 95% credibility level. Incorporating these elk movements between Banff and the Bow did not yield qualitative differences in the graphical distribution of the model.

### Model 5: Short-term, Source-Sink Wolf Movement

Model 5 provided the worst fit of the data among the models by RSS and AICc and received the lowest likelihood weight (0.019) among the candidate set (Table 2) The comparable parameter estimates from Model 5 agreed with the parameter estimates from Models 1, 2, 3 and 4 (Table 1) and the graphical fit of Model 5 did not differ from Model 1. The median wolf encounter rate of Banff elk, *d_1_*, was 0.0007 with a 95% credibility interval of (0.00002, 0.00387) (Table 1). The median encounter rate between wolves and Bow Valley elk, *d_2_*, was 0.02 with 95% credibility interval (0.013, 0.029) (Table 1).

## Discussion

Despite the potential importance of the refuge to predator-prey dynamics in our wolf-elk system, we found little support for interactions between refuge and non-refuge populations through source-sink movements of either prey or predators. Although all models provided approximately the same graphical fit of the data (Fig. 2) with similar parameter estimates (Table 1), Model 1 provided the best fit of the data by AICc and received a substantial portion of the likelihood weight (Table 2). Comparing Models 1, 2 and 4 suggests that elk movement from the Banff refuge to the Bow Valley plays a minimal role in the predator-prey dynamics of BNP, as the median dispersal estimate was only 0.01 of Banff elk/year for both Model 2 and Model 4 (95% credibility intervals: 0.001, 0.037 and 0.001, 0.036, respectively, Table 1). Model 3 received more support than Model 2 (Table 2), but still did not match the support of Model 1, providing little evidence that Bow Valley refuge elk moved to the Banff refuge as a risk avoidance behavior, even though median anti-predator movement estimates had somewhat larger values (median: 0.19 of Bow Valley elk/year; 95% credibility interval: 0.012, 0.775). Model 4 estimated similar rates of anti-predator movements from Bow Valley to Banff (median: 0.18 of Bow Valley elk/year; 95% credibility interval: 0.011, 0.639), but received less support than Models 1, 2 or 3 (Table 2). These results suggest that source-sink movements of prey did not play a role in the predator-prey dynamics of the system. This negative result echoes that of Sepulveda and Lowe [25], who found no evidence of source-sink dynamics between refuge and non-refuge habitats, but contrasts with observations from other harvested ungulate systems [20], [23], [37].

For example, Putman [20] used a simple modeling approach to show that red deer (*Cervus elaphus*) harvest in portions of the study area may only precipitate movement from adjacent areas. Similarly, Naranjo and Bodmer [23] showed red brocket deer (*Mazama americana*) to be subject to unsustainable harvest, yet have higher densities in heavily hunted areas relative to areas with lower hunting pressure, indicating that movement between regions with low and high hunting rates may be crucial to maintaining populations in this species. They also observed low densities with a skew towards young individuals in Baird's tapir (*Tapirus bairdii*) in the high hunting pressure areas, suggesting movements of young, dispersing individuals to hunted sinks [23]. As a contrast, Waber et al. [37] used a model similar to that of Putman [20] to conclude that the observed harvest of Reeve's muntjac (*Muntiacus reevesi*) and roe deer (*Capreolus capreolus*) does not remove a sufficient number of animals to curb dispersal outside of the area considered. In these studies, the nature of predation, harvest by managers and hunters that is limited in spatial and/or temporal extent, differs substantially from the wide-ranging and continuous threat posed by wolves. Moreover, these authors present contrasting directionalities of source-sink movements, as the red deer, red brocket deer and Baird's tapir likely move from areas with lower hunting pressure to actively culled areas, while Reeve's muntjac and roe deer disperse from an actively culled area to the wider landscape. These differences highlight that underlying habitat quality or productivity, in addition to predation, plays a key role in structuring source-sink movements [21], [22]. In BNP, human development increases habitat quality, while simultaneously reducing predation risk in the Banff refuge. These predator and landscape differences may explain some of the divergence between our results and previously reported evidence for source-sink movements in spatially heterogeneous landscapes.

Similarly, we found no evidence to base differential predation of wolves on refuge and non-refuge elk. Model 5 performed the worst of the tested models by our model selection criteria (Table 2). Furthermore, the refuge Banff elk encounter rate with wolves had extremely low estimated parameter values (median: 0.0007; 95% credibility interval: 0.00002, 0.00387), while the non-refuge Bow Valley elk encounter rate with wolves remained unchanged from the other models (median: 0.02; 95% credibility interval: 0.014, 0.029; Table 1). Thus, short-term or facultative wolf movements to depredate Banff refuge elk did not link refuge and non-refuge predator-prey dynamics. In BNP, Banff and the Bow Valley appear to function as separate systems with respect to annual wolf-elk predator-prey dynamics.

Human disturbance may play a key role in maintaining the relative independence of developed refugia from non-refugia in the elk populations of BNP. This disturbance may allow for habitat specialization among populations. The Banff elk may become habituated to development, as both a refuge from predation and a source of high-quality forage, while Bow Valley elk perceive the persistent human activity as a threat [35], [38]. These findings differ from previous work on the forage-risk trade-off that would suggest greater risk avoidance in the refuge where forage resources are readily available [74]. The differences between refuge and non-refuge areas may create specialization in the forage-risk trade-off [75]–[79], which may limit predator and/or prey movements among patches [8], [80]. Indeed, elk movements among regions of BNP were largely temporary [45]. This contrasts with evidence that more direct human intervention, such as harvest, may induce source-sink dynamics [18], [20], [24]. In Banff, human disturbance may provide a stable means of structuring prey populations.

The elk population structure induced by human development may have promoted the persistence of local predator-prey dynamics. In our example, the spatial variation in predation risk was more or less permanent, compared to other systems, where both spatial and temporal variation in predation risk may lead to different relationships between predator and prey populations. This permanent spatial heterogeneity in predation risk may also foster the persistence of spatially distinct prey populations [8], [81]. Since wolves and elk do not apparently move between refuge and non-refuge areas, predator abundance represents a response to local prey resources alone. Human landscape alterations function to decouple predator-prey dynamics, as has been shown for predator-prey dynamics of birds and nest predators in urban areas [82]. Human habitat disturbance may limit predators in the BNP system, while promoting the long-term persistence and stability of prey populations. Human disturbance may structure the landscape, such that wolves neither exploit prey resources of neighboring areas nor produce source-sink dispersal movements among prey populations.

Human disturbance may have had additional influences on the BNP wolf-elk dynamics. Given the varying forage-risk tradeoff of the Banff refuge, elk may not experience density-dependent pressure to disperse until their population approaches or reaches carrying capacity [80]. The Banff refuge population approached these densities at approximately the same time as Parks Canada began translocating elk to mitigate human-wildlife conflicts [51]. This translocation effort may have confounded our ability to detect density-dependent elk dispersal from the Banff refuge to the Bow Valley (Fig. 3). If the Banff elk population continued the trajectory prior to this management action, we may have found more evidence for elk dispersal from Banff to the Bow Valley and source-sink dynamics among refuge and non-refuge elk populations.

Although we found little support for differential predation rates on refuge and non-refuge elk, and in fact the top model fit zero wolf predation to Banff elk, these results conflict with previous empirical work done on wolf-elk dynamics in BNP. Hebblewhite et al. [43] reported a wolf kill rate of 0.17 elk/day/pack for the Bow Valley and a wolf kill rate of 0.06 elk/day/pack for Banff. These numbers suggest that 26% of wolf kills from this system take place in Banff - a stark contrast to the 1.7% estimate from our model. The predation rates of Hebblewhite et al. [43] suggest a greater “spillover” of predators into neighboring areas, as has been observed in a number of predator-prey systems [83]–[86]. These two different measures predict different roles of the Banff elk in the predator-prey dynamics of the Bow Valley.

These conflicting results highlight the possible limitations of the time-series data used in this study. We used annual winter counts of wolves and elk over a 26-year period. Despite the length of our time-series, empirical data often support simpler models over more sophisticated, theoretically sound models, in this case, failing to support the importance of source-sink dynamics [73]. This does not necessarily mean that source-sink dynamics do not occur, ecologically, in our system, particularly if source-sink dynamics vary seasonally [87], occur on short-time scales (e.g., daily) due to temporary shifts in the distribution of prey or predators [88], or individuals moved outside of the study area [22]. Nonetheless, our models suggest that though these finer spatio-temporal source-sink dynamics may occur, they do not measureably alter annual population dynamics.

There may have also been some methodological reasons for concerns over our results. Our choice to describe these data with continuous-time models may have further obscured the predator-prey population dynamics by ignoring the discrete nature of reproduction in these relatively small populations [89], [90]. Even when using the appropriate annual temporal scale, determining the underlying mechanisms behind complex predator-prey dynamics from time-series data is challenging [89], [90]. Thus, even models that show a good fit of the observed population trajectories may only mimic patterns without accurately capturing the true dynamics. Furthermore, we must acknowledge the many theoretical flaws implicit in the Lotka-Volterra model [55]. We may have compounded these model flaws by over-parameterizing the models to assess the multiple potential dynamics of the system. Alternatively, we may have failed to consider important components of predator-prey dynamics in this system, namely the shape and form of the functional response (e.g., [1], [3], [90]), potential time lags in dynamics [4], [56] or temporal variability in parameters [89], [90]. If we had included these (or other) factors in our models, we may have discovered a better fitting model that would suggest more complex source-sink dynamics between the refuge and non-refuge. Nonetheless, the balance between empiricism and theory often yields simpler models than theoretically possible [73], but that still reliably capture salient properties of a system.

Regardless, some support for our parameter estimates, and thus models, comes from comparison to previously published literature. First, both Banff elk population growth rate and carrying capacity were similar to previously published empirical estimates [43]. Second, wolf mortality rate estimates of 0.23–0.25 across models were identical to previous empirical estimates based on radio-collared wolves (annual survival = 0.77) [91]. Also, Vucetich and Peterson [92] and Carbone and Gittleman [93] estimate conversion efficiency for wolves at 1% and 1.2%, respectively, both of which fall below the lower 95% credibility bound of conversion efficiency (Table 1). We expect our modeled estimate of conversion efficiency of just elk to wolves to be biased high since wolves included at least 30% other prey in the Bow Valley [42], [94]. When considering the other prey in wolf diets, our estimated conversion efficiency falls in line with previous studies and lends credence to our general approach, while adding to the limited literature on wolf-prey conversion efficiency.

## Conclusions

Despite potential limitations of our study, the results support that at least on annual time-scales, population dynamics of wolves and elk in the Bow valley were functionally separated and not linked by source-sink dynamics. Application of Occam's razor (that any model should make as few assumptions as possible, eliminating those that have no impact on the observed predictions) to our model set clearly rejects that we need to invoke source-sink dynamics to explain annual predator-prey dynamics of wolves and elk in this system. Predator-prey dynamics play an important role in species management [37], [95], [96], despite the challenges of faithfully describing these complex biological dynamics. Across North America, wolf-prey dynamics have the potential to impact ecosystem structure and function, and present a challenge for ecosystem management. Hebblewhite et al. [39] showed that the high elk density associated with the Banff refuge negatively impacted aspen recruitment, willow production, beaver lodge density, and riparian songbird density and abundance. High elk densities precipitate cascading ecological effects [97]–[101]. These effects may have special import for management of rare or threatened secondary prey species, such as woodland caribou, through predator-mediated apparent competition [102]. Moreover, the effects of refuge elk herds often conflict with the human component of the landscape. Dense urban and suburban elk herds damage property and threaten human health and safety [35], [38]. The impacts of elk refugia require long-term management to maintain ecological diversity and minimize human-wildlife conflicts. Although human disturbance seems to decouple refuge elk populations from the predator-prey dynamics of neighboring areas, these management actions should account for potential interactions between refuge prey and outlying predator-prey dynamics through source-sink movements or other mechanisms.
